# The priority setting of factors affecting a crash severity using the Analytic Network Process

**DOI:** 10.5249/jivr.v12i1.1229

**Published:** 2020-01

**Authors:** Milad Safari, Seyed Shamseddin Alizadeh, Homayoun Sadeghi Bazargani, Atefeh Aliashrafi, Mohammad Shakerkhatibi, Parisa Moshashaei

**Affiliations:** ^*a*^Student Research Committee, Tabriz University of Medical Sciences, Tabriz, Iran.; ^*b*^Department of Occupational Health Engineering, Tabriz University of Medical Sciences, Tabriz, Iran.; ^*c*^Road Traffic Injury Research Center, Tabriz University of Medical Sciences, Tabriz, Iran.; ^*d*^Department of Water Resources Engineering, University of Tabriz, Tabriz, Iran.; ^*e*^Health and Environment Research Center, Tabriz University of Medical Sciences, Tabriz, Iran.

**Keywords:** Traffic accidents, Safety, Injury, Fatality, Iran

## Abstract

**Background:**

The original step in reducing crash severity is recognition of the involved factors. The aim of this paper is to prioritize the factors affecting crashes severity. The current study was carried out in 2018 in Iran.

**Methods:**

The present cross-sectional study focuses on factors affecting the crash severity. Due to the compli-cated nature of traffic accidents, Multi-Criteria Decision-Making methods can be considered as an effective approach. In this work, the factors affecting a crash severity were identified and then attained factors were scored by ten traffic safety experts. To prioritize and weigh these factors, the Analytic Network Process method and Super Decisions program were used.

**Results:**

The results showed four main factors and 60 sub-factors in which the main factors in the order of priority were the safety (the most important sub-factor: speed over the upper limit), the other fac-tors (the most important sub-factor: road user type), the health (the most important sub-factor: drowsiness), and the environment (the most important sub-factor: slipping the road).

**Conclusions:**

In order to control the crash severity, the presented factors in this study could help traffic safety experts to prioritize and perform controlling actions.

## Introduction

In the light of modernized communities and the integrity of motor vehicles with human lifestyle, land transporta-tion has been at the edge of a great evolution during the recent century.^1^ Increasing the number of cars, growing the traffic volume on the roads and the lack of safety have raised up the incidence and severity of traffic accidents.^2^ Traffic accident is defined as collision of a vehicle with another one, a human, an animal, or other motionless objects,^3^ in which its most serious outcomes are the injuries and mortality.^4^ The analysis of resulted costs from road accidents re-vealed that the economic burden of traffic accidents is about 1% to 2% of gross national product (GNP) among developing countries.^5-7^ A recent study disclosed that all costs of road traffic accidents (RTAs) in Tehran province are about 3% to 4% of gross domestic product (GDP) of Iran in the same year.^8^

Nearly 1.3 million subjects demise due to the RTAs annually, and it is estimated that the number will increase to 1.9 million subjects by the end of 2020.^9^ The injuries and mortality of traffic accidents are overwhelmingly rising in developing countries.^10^ According to WHO (2018), road traffic resulted the death of almost 16000 people in Iran. ^11^ Therefore, the consequences of these crashes are signifi-cant concerns for the government. ^11,12^

The most important factors including the human, vehicle, road and the environment can influence the severity of road accidents. Kaplan and Prato (2012) showed that the bus drivers under 25 and over the age of 65, female drivers, and risky driving increase the crash severity.^13^ Previous studies disclosed that male drivers significantly affect the traffic violations and crash severity.^14-18^ The vehicle characteristics (i.e., vehicle age, vehicle type, etc.) can also affect the crash severity. Zhang et al. (2013) found that goods vehicles strongly increase the risk of crash severity while, vehicle safety condition checking specifically for goods vehicles, significantly associated with reducing traffic violations, serious injury, and fatality.^19^ According to the literature, on the other hand, the road characteristics associated with an increment of the crash severity. Wang et al. (2017) disclosed that the crash severity is highly impacted with factors such as rural roadway, curvy road, dry roadway conditions, driving during nighttime when the street has a poor lightning condition.^20^ Time and environmental features can be considered as other potential risk factors affecting the traffic crash severity. Ma et al. (2016) showed that factors like season and time of day are in close relation with the severity of injury.^21^ Some previous studies disclosed that adverse weather conditions like rain, snow, and fog led to augmented driving hazards.^22-24^

Reducing the severity of traffic accidents requires identifying the influence of the most involved significant factors, so the aim of this study is to prioritize the factors affecting the crash severity using Analytical Network Process (ANP) techique.

## Materials and Methods

**Identification and validation of factors**

Factors affecting the crash severity were found through a comprehensive literature review using electronic databases including Science Direct, Web of Science and, Google Scholar. The following key words were used to find eligible studies: fatality, road accident, crash, and injuries. As an evaluation of factors’ validity, comments of 20 safety experts were perceived and examined. In this study, the means of the validation were true or not true factors.

**Checklist development and administration**

All factors were collected and approved and then, a checklist was constructed and sent to 10 traffic safety experts to pairwise comparison between main factors and sub-factors. Initially, the main factors were regarding independence, together with the pairwise comparison. Then, sub-factors for each main factors were compared pairwise. Table 1 presents the scale of relative importance for pairwise comparison. The ANP method was used to prioritize the factors and sub-factors.

**Table 1:** The scale of relative importance.^29^

Intensity of importance	Definition
1	Equal importance
3	Moderate importance
5	Strong importance
7	Very strong or demonstrated importance
9	Extreme importance
2,4,6,8	Intermediate values

**ANP technique**

The ANP is a developed form of Analytical Hierarchy Process (AHP), and can be utilized to solve most intricate decision problems.^25^ It is an applicable technique for treating a complicated problem by considering the interdependency among the criteria.^26^ In comparison with the other decision-making models, ANP is capable of taking into account the whole criteria by a similar unit and likewise of other Multi-Attribute Decision-Making (MADM) methods, it is able to examine both quantitative and qualitative states. In comparison with AHP, the ANP is more powerful, due to its capability of considering network associations through the modeling process.^27^ This technique fundamentally provides an intelligent tool for solving decision-making problems. Additionally, the ANP conceptualizes the problem using a network of alternative and criteria. In fact, all criteria in the system can be correlated in any possible way either within or among the cluster. Thus, an exact modeling tool for complete setting and reciprocal dependency among the criteria is presented.^28^

The technique basically includes of four main steps: 

1) Modeling of factors affecting the crash severity based on the included ones. In this way, four main factors as nodes within a cluster were made and the relationships were investigated.

2) Arranging a pairwise comparison matrix and asking the decision maker for estimating the relative significance weights of factors.

3) Supermatrix formation: in this study, the construction of the supermatrix was done using the following command: Computations → Limit Matrix → Graphical

4) Synthesis &sensitivity: the synthesized priorities of alternatives were acquired with the synthesis command. The related definition of each column are as follows:

• The Normals column: presenting the results in the format of priorities which is the common method of reporting results.

• The Ideals column:

**Figure:**
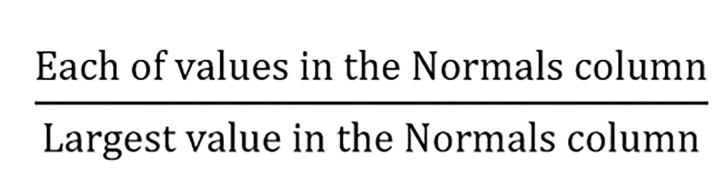


the more significant choice has a priority of 1.

• The Raw column: values is visible from the Limit Supermatrix. In this study, the Raw and Normals columns are the same since it’s a hierarchical model.

The sensitivity analysis is utilized to analyze how the priorities of alternative solutions alter as we change the priority of one or more decision making factors. The sensitivity rate is acceptable if it was less than 0.1.

5) Ranking determination, regarding the alternatives and selection of the best one from the Limit Supermatrix: alternatives were prioritized regard to their weights so that the selected alternative possed the highest weight as the best alternative.^30,31^

The apply ANP method for finding the answer of practical problems is complicated and demands the application of a particular calculation program. Therefore, in the current work, super decisions program (SDP) version 2.0.8 has been applied to prioritize the factors. 

**Super Decisions program**

Super Decisions is a program developed by Creative Decisions Foundation. This program offers tools of making and managing AHP and ANP models. The SDP is applied in decision-making with dependence and feedback.^32^

The ANP is carried out in the SDP and has been used in different decision problems. It is a coupling of two parts. The first includes a control hierarchy or network of factors and sub-factors that control the reciprocal actions of understudy system. The second one is a network of effects among the elements and clusters. Applications may be uncomplicated, include a single network, or complicated, and include the principal network and two or more layers of sub-networks. Each network and sub-network is formed in its own window.^33^

## Results

According to the literature review, we obtained 60 sub-factors that two of them were eliminated based on the comments of experts. As can be seen in Table 2, the rest of the sub-factors were classified into four factors. 

**Table 2:** Categorizing the sub-factors affecting the crash severity.

No.	Factor	Frequency of sub-factors	Sub-factor
1	Health	6	Consumption of alcohol^34^ Drug use^35^ Drowsiness^36^ Fatigue^36^ Impairment^37^ Distraction^37^
2	Safety	25	Speed over upper limit,^38^ Failure to use seat belt,^39^ Improper overtaking, ^40^ Turning violation,^40^ Failure to comply with the longitudinal distance, ^41^ Failure to comply transverse distance,^41^ Not paying attention to the front, ^41^ Lack of skill in driving and controlling a vehicle,^41^ Changing direction suddenly, ^41^ Failure to comply with priority, ^41^ Phoning,^42^ Kilometres travelled by vehicles,^43^ Mechanical defects,^44^ Having an ABS and ESP system, ^43^ Vehicle type,^38^ Vehicle overload condition,^19^ Weight of vehicle,^44^ Tire defect,^40^ Airbag, ^45^ Compulsory third party insurance,^19^ Trapping inside vehicle,^46^ Ejection out of vehicle,^46^ Fire following collision,^46^ Position in vehicle^45^ Congested road^44^
3	Environment	22	Lighting conditions,^47^ Bad visibility (Night, Sunset, Sunrise), ^48^ Crash time,^48^ Working days,^43^ Weekend and public holidays, ^43^ Month, ^19^ Season,^19^ Weather condition,^47^ Slipping the road,^47^ Temperature,^49^ Wind speed (km/h),^49^ Road surface conditions (Dry, Wet, Snowed, Iced),^47^ Quality of road asphalt,^38^ Road class type (Local city street, Highway, Provincial road, Public vehicular area),^38^ Traffic-way (One-way and Two-way),^13^ Number of lanes per direction, ^13^ Road alignment (Straight, Curve),^13^ Road profile (Level, Grade),^13^ Raised median, ^50^ Painted median,^50^ Near tunnel entrance/ exit,^21^ Tunnel length.^21^
4	Others	7	Point of collision (Head-on, Rear end, etc.),^47^ Number of vehicles involved in the accident,^47^ Purpose of use vehicle (Commercial vehicle, Private vehicle), ^38^ Crash location (Not at intersections, At intersections, etc.),^48^ Crash type (Single vehicle, Multiple vehicles, Pedestrians),^47^ Road users involved (Pedestrian, Bicycle, Heavy vehicles, Moped, Car, Animal),^48^ Distance from a hospital. ^48^

The following steps demonstrate the prioritization of factors and sub-factors using SDP.

***Step 1: Constructing clusters and making relationships between them***

**Figure 1:**
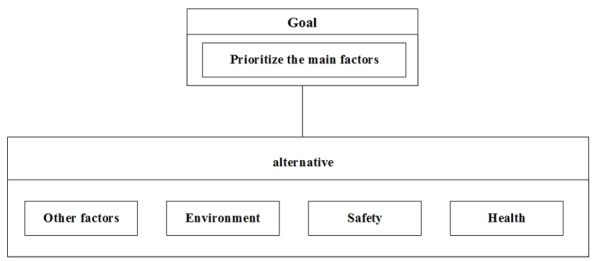
The main factors model.

Figure 1, indicates the results acquired from the SDP. In a similar manner, node, cluster and the internal relationship among them were formed exclusively for each sub-factor.

***Step 2: Pairwise comparisons of factors and sub-factors ***

There are four pairwise comparison assessment methods among which the questionnaire method was selected. In this segment, pairwise comparison questionnaire was perfected based on the experts’ approach who had sufficient experience in the analysis of crash severity.

***Step 3: Creation of super-matrix from factors and sub-factors priority***

According to the supermatrix (Table 3), the safety and environment have the highest and lowest values, respectively. Similarly, the super-matrix for each of the sub-factors was formed.

**Table 3:** Limit Matrix for the main factors.

Cluster Node Labels		Alternative				Goal
		Environ-ment	Health	Other fac-tors	Safety	Prioritize the main criteria
Alternative	Environment	0.000000	0.000000	0.000000	0.000000	0.113122
	Health	0.000000	0.000000	0.000000	0.000000	0.122172
	Other factors	0.000000	0.000000	0.000000	0.000000	0.130090
	Safety	0.000000	0.000000	0.000000	0.000000	0.634615
Goal	Prioritize the main criteria	0.000000	0.000000	0.000000	0.000000	0.000000

***Step 4: Synthesis & sensitivity***

In this study, the sensitivity rate is less than 0.1 for factors (0.0068) and sub-factors (0.0565). But as shown in Table 4 in the “Raw” and the “Normals” columns this value is different. In current study, the final weight of main factors and sub-factors for crash severity is expressed according to “Normals” column. Table 4 presents the results of synthesis rate for the main factors. As shown in this table, the safety factor has the highest priority followed by the other factors, health and the environment factors, respectively. Also, Table 5shows the final weight of main factors and sub-factors for crash severity.

**Table 4:** Main factors synthesis.

Factor	Ideals	Normals	Raw
Environment	0.178253	0.113122	0.113122
Health	0.192513	0.122172	0.122172
Other factors	0.204991	0.130091	0.130091
Safety	1.000000	0.634616	0.634616

**Table 5:** Weight prioritization main factors and sub-factors for crash severity.

Rank	Main factors	Weight obtained(%)	Rank	Sub-factors	Weight obtained(%)	Sensitivity rate
1	Safety	63	1	Speed over upper limit	7.0	0.048
			2	Lack of skill in driving and controlling a vehicle	6.5	
			3	Change direction suddenly	6.1	
			4	Phoning	5.6	
			5	Failure to comply with priority	5.5	
			6	Not paying attention to the front	5.1	
			7	Having an ABS and ESP system	5.0	
			8	Fire following collision	5.0	
			9	Improper overtaking	4.9	
			10	Mechanical defects	4.8	
			11	Ejection out of vehicle	4.4	
			12	Failure to comply with the longitudinal distance	4.4	
			13	Airbag	4.3	
			14	Tire defect	4.2	
			15	Failure to use seat belts	3.7	
			16	Trapping inside	3.4	
			17	Turning violation	3.3	
			18	Vehicle type	3.0	
			19	Failure to comply transverse distance	2.5	
			20	Position in vehicle	2.2	
			21	Congested road	2.0	
			22	Compulsory third party insurance	1.8	
			23	Kilometres travelled by vehicles	1.5	
			24	Vehicle overload condition	1.4	
			25	Weight of vehicle	1.3	
2	Other factors	13	1	Road users involved (Pedestrian, bicycle, Heavy vehicles, Moped, Car, Animal)	18.0	0.056
			2	Number of vehicles involved in the accident	17.2	
			3	Crash type (single vehicle, multiple vehicles, pedestrians)	17.0	
			4	Distance from a hospital	14.9	
			5	point of collision (head-on, rear end, etc.)	14.1	
			6	Crash location (not at intersections, at intersections, etc.)	13.0	
			7	Purpose of use vehicle (Commercial vehicle, Private vehicle)	4.3	
3	Health	12	1	Drowsiness	26.0	0.047
			2	Impairment	23.0	
			3	Fatigue	19.0	
			4	Consumption of alcohol	11.0	
			5	Distraction	10.0	
			6	Drug use	8.0	
4	Environment	11	1	Slipping the road	12.0	0.052
			2	Road surface conditions (Dry, Wet, Snowed, iced)	7.4	
			3	Traffic-way (One-way and Two-way)	6.9	
			4	Weather condition	5.8	
			5	Road alignment (Straight, Curve)	5.7	
			6	Road class type (local city street, highway, provincial road, public vehicular area)	5.2	
			7	Raised median	5.1	
			8	Number of lanes per direction	4.6	
			9	Bad visibility (Night, sunset sunrise)	4.5	
			10	Quality of road asphalt	4.4	
			11	Lighting conditions	4.3	
			12	Painted median	4.1	
			13	Near tunnel entrance/ exit	3.9	
			14	Road profile (Level, Grade)	3.9	
			15	Crash time	3.7	
			16	Tunnel length	3.3	
			17	Weekend and public holidays	2.8	
			18	Temperature	2.6	
			19	Working days	2.2	
			20	Month	2.1	
			21	Season	2.0	
			22	Wind speed (km/h)	1.8	

## Discussion

Based on the weights, major factors were the safety, other factors, health, and the environment. In the following, factors and the most significant sub-factors are explained in the order of preference.

***Safety factors***

As shown in Table 4, safety is allotted the highest rank in the factors affecting crash severity. The crash severity can be adversely affected by the safety factors such as over limited speed, lack of driving skill and controlling ability of vehicles, sudden change of directions, etc. Among the safety factors, speed over upper limit sub-factor had the highest priority. This result is consistent with the previous studies.^47,51,52,53^ According to Kadilar (2016), the speed has an important role on the severity of crashes so that the risk of driving at the high speeds (> 111km/h) is three times more than driving at speeds of<56 km/h.^35^

***Other factors***

The second rank of factors affecting the crash severity is other factors. The crash severity can be unfavorably affected by other factors such as road users involvement (pedestrian, bicycle, heavy vehicles, moped, car, an animal), the number of vehicles involved in the accident, crash type (single vehicle, multiple vehicles, pedestrians), etc. The road user type has the highest rank among other factors in crash severity. Prato et al. (2014) asserted that nearly one-quarter of the traffic accidents involved susceptible road users. The most unpleasant outcomes are related to the motorcyclists and pedestrians, who are associated with 140% higher likelihood to tolerate serious injuries and 240% to 260% higher likelihood to fatal.^48^ Ma et al. (2015) found that crashes associated with pedestrian are probable of bearing more severe injuries.^51^ Also, other studies referred to this issue.^54,55^

***Health factors***

Health factors could have a massive negative influence on the rise of resulted fatal and injuries from the road accidents. One of the most significant health sub-factor is drowsiness. National Highway Traffic Safety Administration (NHTSA) estimates that the number of drowsy drivers involved in crashes was 90,000 among police-reported accidents in 2015. These crashes led to an almost 41,000 people injures and more than 800 deaths.^56^ Sleepiness and the need to sleep while driving can be attributed to several causes: 1) lack of sleep 2) job-related sleep restriction 3) personal demands and lifestyle choices 4) sleep fragmentation 5) circadian factors. ^57^ Abegaz et al. (2014) found that falling asleep while driving (Coef: 1.3102; p-value<0.001) significantly associated with an increment of the severity of crash injury.^42^

***Environmental factors***

Environmental factors such as slippery road, road surface conditions (dry, wet, snowed, iced), traffic-way (one-way and two-way), etc. have an essential impact on crash severity. Findings show that among the environmental factors, slippery road sub-factor had a higher priority. Additionally, studies in Finland indicated that the underlying reason for 47% of fatality was high or moderate speed on slippery road surfaces, inspite the lack of alcohol usage.^58^ Hence, the risk of a slippery road should be reduced by developing specific training programs for drivers. ^59^

***Recommendations***

This study proposes the following strategies for lessening the crash severity proportional to the above factors:

• There are various methods contributed in diminishing vehicle speeds, including legislation, road design, and severe enforcement (e.g., speed cameras). One opinion is Intelligent Speed Adaptation (ISA), a system in which the vehicle 'knows' the speed limit of road driving on, and can activate visual and audio signals in the case of passing the threshold. ^60^

• Enhance the awareness of susceptible road users and improve their safety in traffic conditions.^48^

• Cease to breaks taking every two hours or 100 miles during long trips and avoid alcohol drinking if you feel fatigued since it has a close relation with the degree of drowsiness. ^61^

• Regular road inspection. 

• Remove the slider as much as possible.

• Slow down speed on slippery roads.

***Study limitation***

The main crisis of the current study was difficult access to traffic safety experts.

## Conclusion

In this study, priority setting of the factors affecting the crash severity was put forward. The relationship between four main factors and 60 sub-factors effective in the crash severity was evaluated. The findings indicate and categorize the importance of each factor (from high to low) in increment of crash severity as follow: safety (the most important sub-factor: speed over upper limit), other factors (the most important sub-factor: road user type), health (the most important sub-factor: drowsiness) and the environment (the most important sub-factor: slipping the road). This paper will assist in the recognition of involved factors in crash severity. That way, we can prioritize and execute monitoring and also preventing processes for moderating the severity of crashes.

**Acknowledgements**

We thank all of the experts for the plentiful support to the study.
